# Durable Complete Response Following Nivolumab Discontinuation in Human Papillomavirus (HPV)-Positive Oropharyngeal Carcinoma Complicated by Bullous Pemphigoid: A Case Report

**DOI:** 10.7759/cureus.112740

**Published:** 2026-07-15

**Authors:** Maria Abreu, Vanessa Branco, Filipa Ferreira, Ana Martins

**Affiliations:** 1 Oncology, Unidade Local de Saúde Lisboa Ocidental, Lisboa, PRT; 2 Medical Oncology, Unidade Local de Saúde Lisboa Ocidental, Lisboa, PRT

**Keywords:** case report, durable response, head and neck cancer, hpv, immune-related adverse events, immunotherapy, nivolumab, oropharyngeal carcinoma

## Abstract

Immune checkpoint inhibitors have improved outcomes in recurrent/metastatic head and neck squamous cell carcinoma (HNSCC), although durable complete responses (CRs) remain uncommon. The optimal duration of immunotherapy is not yet established.

We report the case of a 69-year-old female smoker diagnosed in November 2015 with human papillomavirus (HPV)16-positive squamous cell carcinoma of the oropharynx (base of tongue), staged cT2N2bM0. Following induction chemotherapy with docetaxel, cisplatin, and 5-fluorouracil, and concurrent chemoradiotherapy with cisplatin, persistent disease was confirmed by biopsy. In December 2016, she underwent extensive surgery that included partial pharyngectomy with tracheostomy + hemiglossectomy + bilateral neck dissection + microvascular free flap reconstruction with positive margins (ypT4aN2R1). Following surgery with positive margins, complementary systemic therapy was administered (paclitaxel, followed by cetuximab combined with methotrexate). In 2019, the patient presented with locoregional cutaneous recurrence, which required hemostatic palliative radiotherapy. Nivolumab was initiated in December 2019, and a CR was achieved about two months later, by February 2020. Treatment was discontinued in February 2021 due to immune-related toxicity (bullous pemphigoid, Common Terminology Criteria for Adverse Events v5.0, grade 3). Remarkably, the patient has maintained a CR for more than four years after discontinuation of immunotherapy, as confirmed by imaging in 2025.

This case highlights the potential for durable CRs after immunotherapy discontinuation in heavily pretreated HPV-positive HNSCC, raising important questions regarding optimal treatment duration, patient selection, and the role of immune-related adverse events as a potential marker of response.

## Introduction

Head and neck squamous cell carcinoma (HNSCC) remains a challenging disease in the recurrent or metastatic setting, with limited long-term survival. The introduction of immune checkpoint inhibitors, such as nivolumab and pembrolizumab, has improved outcomes in this patient population; however, overall response rates remain modest, ranging between 13% and 20% in pivotal trials, and complete responses (CRs) are rare [1-3]. Long-term follow-up data have confirmed a five-year overall survival of approximately 19% in patients with recurrent/metastatic HNSCC treated with nivolumab [4]. Nivolumab is an anti-programmed cell death (PD)-1 monoclonal antibody that blocks the interaction between the PD-1 receptor on T lymphocytes and its ligands PD-L1/PD-L2, thereby restoring antitumor immune activity.

Human papillomavirus (HPV)-associated oropharyngeal carcinoma exhibits distinct biological and immunological features, including higher CD8+ T-cell infiltration and elevated PD-L1 expression, and is associated with a better prognosis compared with HPV-negative disease, although significant variability in treatment response persists [5,6]. Importantly, the optimal duration of immunotherapy for patients who achieve a response is not well established and remains an area of active clinical investigation. Recent data suggest that the progression-free survival curve plateaus at three years in long-term nivolumab responders, pointing toward the possibility of safe discontinuation in selected patients [7]. This distinct molecular profile, including persistent expression of the viral oncoproteins E6 and E7 and a more immunogenic tumor microenvironment, has been proposed to underlie the more favorable treatment response observed in HPV-driven disease [8]. Furthermore, HPV-related oropharyngeal carcinoma represents a substantial and increasing proportion of head and neck cancers in several populations, underscoring its epidemiological relevance [9].

We report the case of a patient with HPV-positive oropharyngeal carcinoma, treated with multiple therapeutic lines, who achieved a durable CR after nivolumab discontinuation, sustained for more than four years.

## Case presentation

A 69-year-old female smoker with a history of duodenal ulcer and regular medication including omeprazole, simvastatin, amlodipine, and valsartan was referred in November 2015 for neoadjuvant chemotherapy. She presented with a foreign body sensation in the hypopharynx lasting approximately 10 days, previously treated with amoxicillin/clavulanate with modest improvement. She denied dysphagia, dysphonia, dyspnea, or odynophagia. Physical examination revealed a hypertrophic lesion at the left base of the tongue, firm on bimanual palpation, and bilateral laterocervical lymphadenopathy, more prominent on the left (level III). Indirect videolaryngoscopy showed a lesion involving the left half of the base of the tongue/vallecula, without apparent extension to the tonsil or lateral pharyngeal wall. The patient has an Eastern Cooperative Oncology Group Performance Status of 1 and weighs 56 kg.

**Figure 1:**
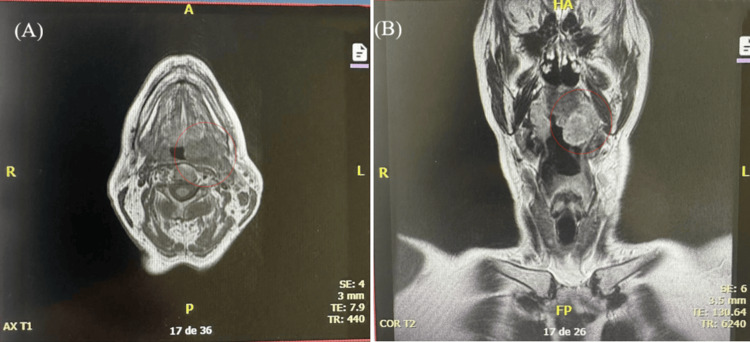
MRI of the oropharynx at diagnosis showing left base of tongue lesion (A) Axial T1-weighted image demonstrating a predominantly exophytic space-occupying lesion at the left base of tongue (circle), measuring 31 × 23 × 22.5 mm, without a cleavage plane with the left tonsil and extending inferiorly to the vallecula. (B) Coronal T2-weighted image illustrating the craniocaudal extent of the primary tumor (circle) and its relationship with adjacent oropharyngeal structures, including the left tonsillar region and the vallecula MRI: magnetic resonance imaging; TE: echo time; TR: repetition time; SE: spin echo

Cervical magnetic resonance imaging (MRI) demonstrated an ovoid space-occupying lesion at the left base of tongue measuring 31 × 23 × 22.5 mm (craniocaudal (CC) × anteroposterior × transverse), predominantly exophytic, without a cleavage plane with the left tonsil and extending inferiorly to the vallecula (Figure 1).

There was no apparent involvement of the ipsilateral glossoepiglottic fold or posterior pharyngeal wall. A conglomerate lymph node mass was identified at the left high internal jugular chain (levels II and III), with a CC extension of approximately 5 cm comprising at least four lymph nodes. No supraclavicular lymphadenopathy was documented.

Biopsy revealed a well-differentiated, ulcerated squamous cell carcinoma with marked inflammatory infiltrate and P16 positivity. The case was discussed at a multidisciplinary tumor board and staged as cT2N2bM0, HPV16-positive. Induction chemotherapy followed by concurrent chemoradiotherapy was unanimously recommended.

The patient completed three cycles of modified docetaxel, cisplatin, and 5-fluorouracil induction chemotherapy and subsequently underwent concurrent chemoradiotherapy with cisplatin (from February to April 2016). The patient received a dose of 59.4 Gy to the left cervical lymph node levels and 69.6 Gy to the primary tumor lesion, in 33 fractions of 1.8 and 2.12 Gy/fraction/day, respectively.

Posttreatment reassessment by cervical MRI (October 2016) was suggestive of persistent disease at the base of the tongue with left cervical lymphadenopathy. Biopsy confirmed persistent squamous cell carcinoma. In December 2016, the patient underwent extensive surgery, including partial pharyngectomy with tracheostomy, hemiglossectomy, and microvascular flap reconstruction. Bilateral neck dissection was also performed: on the right side, a selective neck dissection encompassing levels I, IIa, IIb, III, and IV was performed, with preservation of the internal jugular vein, sternocleidomastoid muscle, and hypoglossal nerve (cranial nerve XII); on the left side, a radical neck dissection was performed, which by definition encompasses levels I-V, together with excision of the internal jugular vein, sternocleidomastoid muscle, and hypoglossal nerve. Pathological examination revealed moderately to poorly differentiated squamous cell carcinoma of the oropharynx (base of tongue) with invasion of adjacent structures, including the larynx, bilateral nodal metastases, and positive surgical margins, staged ypT4aN2R1.

Following surgery with positive margins, complementary systemic therapy was administered. She was first treated with paclitaxel every 21 days (from February to June 2017), but intolerable toxicity ensued, and she then received cetuximab combined with methotrexate every 14 days from June 2017 to August 2019. The patient was maintained under regular cervicothoracic CT surveillance, which excluded distant metastatic disease throughout follow-up.

**Figure 2:**
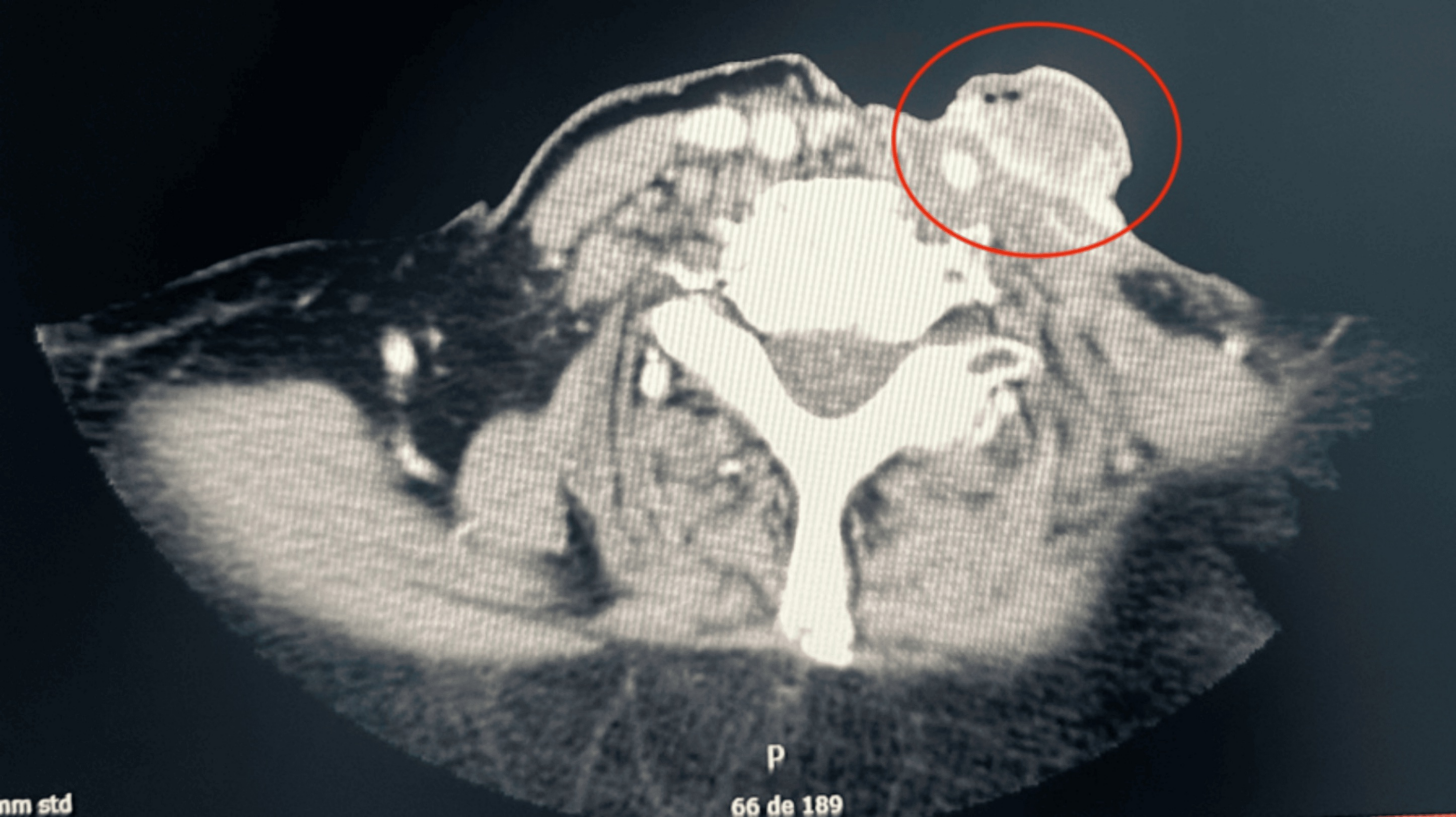
Axial contrast-enhanced cervical CT (August 2019) demonstrating an ulcerated cutaneous lesion of the left anterolateral neck (greatest dimension ~35 mm), consistent with locoregional recurrence CT: computed tomography

In August 2019, cervical and thoracic CT documented a partially ulcerated cutaneous cervical recurrence on the left anterolateral neck, measuring 35 mm in its largest axis (increased from 25 mm in January 2019), with no pathological cervical lymphadenopathy and no evidence of distant metastatic disease, confirming locoregional progression (Figure 2).

In October 2019, hemostatic palliative radiotherapy was administered to the ulcerated, fistulized cervical lesion. Palliative radiotherapy was administered to the exophytic tumor lesion on the left side of the neck in October 2019, delivering a total dose of 30 Gy using 6 and 15 MV photon beams in nine fractions.

After that, following the approval and reimbursement of nivolumab for patients with platinum-refractory recurrent or metastatic HNSCC, immunohistochemical analysis was performed, demonstrating PD-L1 expression (22C3 antibody, DAKO (Agilent Technologies, Santa Clara, US), Ventana platform (Roche Diagnostics, Basel, Switzerland)) with positivity in approximately 1%-2% of viable tumor cells.

Nivolumab was initiated in December 2019, administered every 14 days for eight cycles, subsequently spaced to every 28 days for nine cycles (17 cycles in total). The first on-treatment cervicothoracic CT (February 2020) demonstrated the disappearance of the previously documented left anterolateral cervical soft-tissue lesion, representing the first objective radiological response (approximately two months after initiation) that was maintained throughout treatment.

In October 2020, the patient developed a generalized pruritic rash that was difficult to manage, which prompted referral to a dermatology specialist. The rash partially responded to alternating tacrolimus and hydrocortisone ointments, ebastine, and a short course of tapering prednisolone. The diagnosis of bullous pemphigoid was confirmed by skin biopsy, which demonstrated a subepidermal blister rich in eosinophils, consistent with bullous pemphigoid. Direct immunofluorescence showed linear deposits of C3 and IgG along the dermoepidermal junction. Indirect immunofluorescence and circulating anti-BP180/BP230 antibody testing were not performed. The event was graded as grade 3 according to Common Terminology Criteria for Adverse Events (CTCAE) v5.0 and resolved completely only after nivolumab discontinuation [10].

Cervicothoracic computed tomography (CT) in January 2021 documented a CR with no evidence of local or distant disease: no soft-tissue lesions or anomalous enhancement at the site of prior glossopharyngectomy, no cervical nodal recurrence bilaterally, and no pulmonary nodules suggestive of secondary deposits.

**Figure 3:**
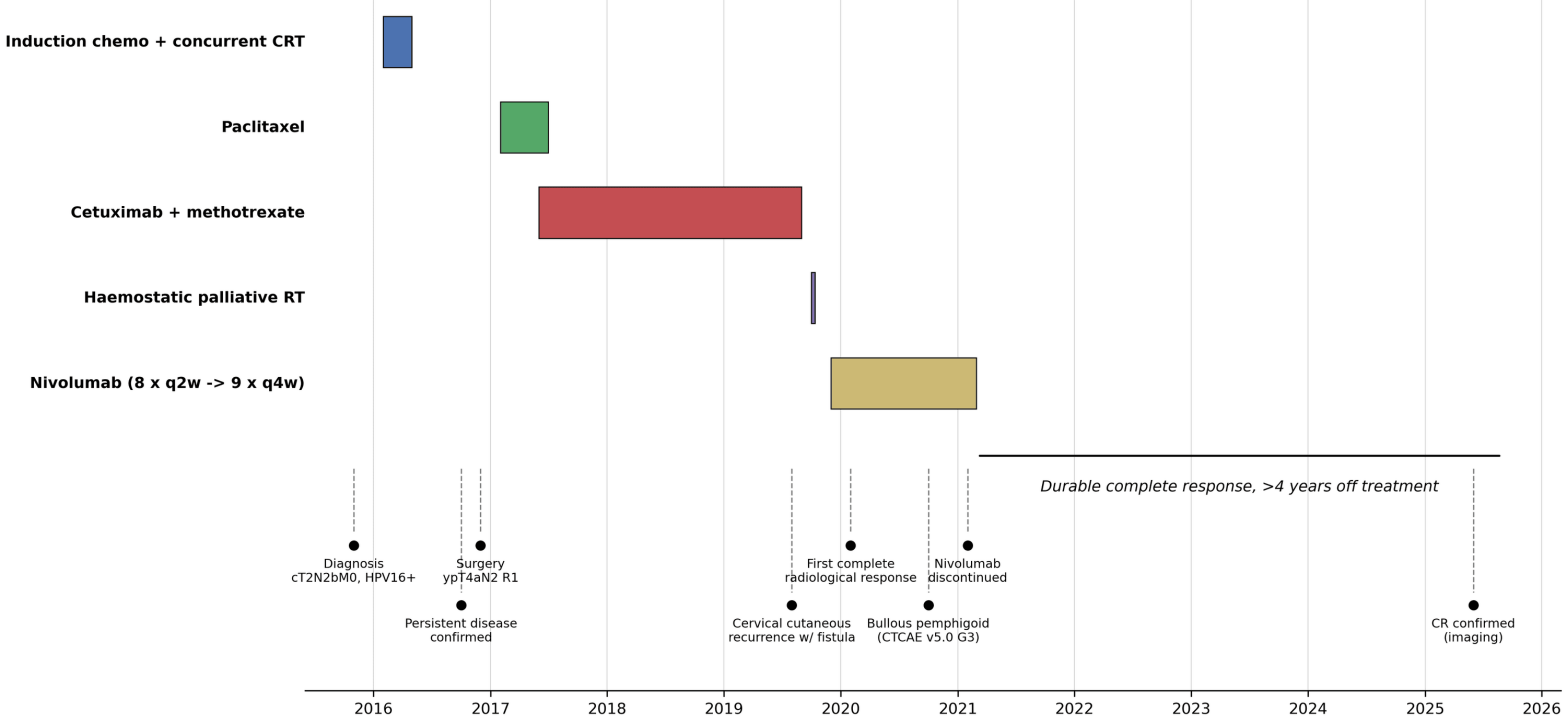
Swimmer-plot timeline summarizing treatment lines, clinical events, and the durable CR Colored bars represent the systemic and radiotherapy treatment lines administered throughout the disease course: induction chemotherapy with concurrent chemoradiotherapy (February-April 2016), paclitaxel (February-June 2017), cetuximab combined with methotrexate (June 2017-August 2019), hemostatic palliative radiotherapy (October 2019), and nivolumab (December 2019-February 2021; eight cycles every 14 days followed by nine cycles every 28 days). Markers on the lower axis denote key clinical and radiological events, including diagnosis (cT2N2bM0, HPV16-positive), confirmation of persistent disease, surgery (ypT4aN2 R1), cervical cutaneous recurrence with fistulization, the first documented complete radiological response (February 2020), bullous pemphigoid as an immune-related adverse event (CTCAE v5.0 grade 3), and nivolumab discontinuation (February 2021). The CR has been sustained for more than four years off treatment, last confirmed by imaging in June 2025 CT: chemotherapy; CRT: chemoradiotherapy; RT: radiotherapy; HPV: human papillomavirus; CTCAE: Common Terminology Criteria for Adverse Events; CR: complete response

Given the durable CR to immunotherapy, clinically significant immune-related toxicity (bullous pemphigoid, G3), and significant logistical challenges in attending the hospital, nivolumab was discontinued in February 2021, with ongoing surveillance. Figure 3 provides a chronological overview of the diagnostic, therapeutic, and response milestones throughout the disease course.

**Figure 4:**
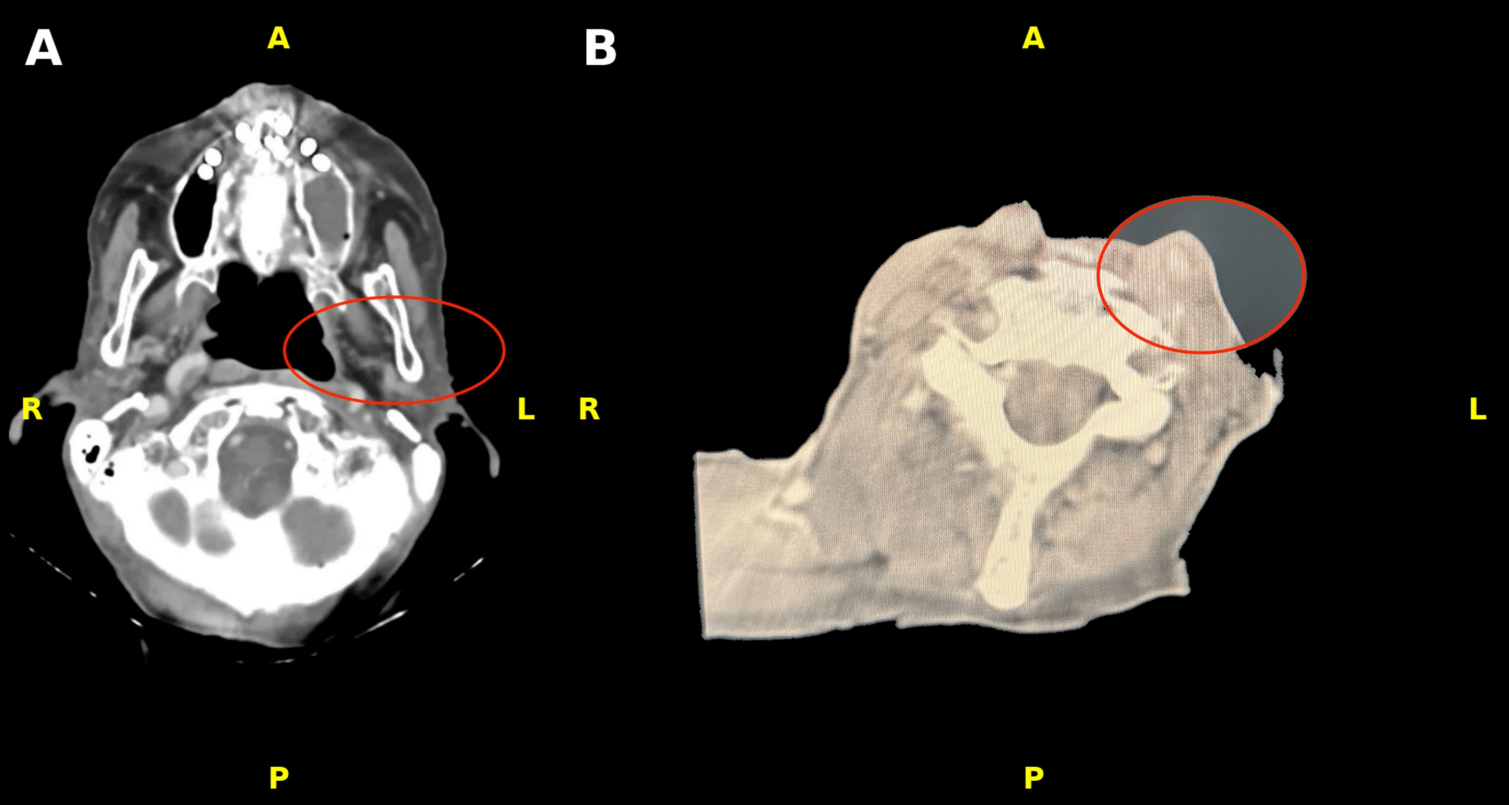
Follow-up CT of the neck (June 2025) demonstrating a maintained complete response (A) Axial section at the level of the prior region of the primary lesion. (B) Axial section at the level of the previous recurrent cervical lesion. In both panels, the red ellipse delineates the site of the original lesion. No residual soft-tissue mass, anomalous contrast enhancement, or pathological lymphadenopathy is identified within these regions or bilaterally, confirming the absence of local or nodal recurrence. Postsurgical changes, tracheostomy, and cervical fibrosis are noted CT: computed tomography

Remarkably, the patient has maintained a CR for more than four years after treatment discontinuation. The most recent clinical evaluation (February 2026) showed an ECOG PS of 2, cachexia (weight 39.5 kg), tracheostomy dependence, percutaneous endoscopic gastrostomy feeding, significant bilateral cervical fibrosis, and a small healed submandibular erosion. Despite these limitations, the patient remains well-adapted to her condition, is functionally independent in activities of daily living, and reports a good quality of life. Laboratory tests and cervicothoracic CT from June 2025 (Figure 4) confirmed the absence of recurrence.

The patient remains under annual follow-up in Medical Oncology, Otorhinolaryngology, Dermatology, and Chronic Pain clinics.

## Discussion

This case is particularly noteworthy for several reasons. First, the patient had been heavily pretreated, having undergone induction chemotherapy, concurrent chemoradiotherapy, extensive surgery with positive margins, and multiple lines of palliative systemic therapy before initiating immunotherapy. Achieving a CR in this context is exceptional, given that overall response rates to nivolumab in recurrent/metastatic HNSCC range from 13% to 20% [1-3].

Second, PD-L1 expression was low (1%-2% of neoplastic cells), a factor typically associated with reduced likelihood of response to immune checkpoint inhibitors. Despite this, a sustained CR was achieved, suggesting that other immunological mechanisms may have contributed to tumor control.

A particularly relevant aspect of this case is the durability of response following immunotherapy discontinuation. The optimal duration of immune checkpoint inhibitor therapy in HNSCC remains unclear, and current clinical practice varies widely. Recent data from Matsuo et al. demonstrated, in a cohort of 246 patients with HNSCC treated with nivolumab, that the progression-free survival curve plateaus completely at three years, suggesting that patients maintaining response beyond this period may benefit from treatment discontinuation [7]. Furthermore, the five-year follow-up of this cohort revealed a five-year overall survival of 19.2% and a median disease-free survival of 33.2 months among patients who discontinued nivolumab due to immune-related toxicity [4]. The present case reinforces these findings, demonstrating that sustained immune-mediated tumor control may persist even after treatment cessation, with CR maintained for more than four years.

The occurrence of bullous pemphigoid as an immune-related adverse event also merits attention. A comprehensive meta-analysis by Zhou et al., including 30 studies and 4,971 patients, demonstrated that patients who developed immune-related adverse events experienced significant benefits in both overall survival (HR, 0.54; 95% CI, 0.45-0.65) and progression-free survival (HR, 0.52; 95% CI, 0.44-0.61) [11]. Cook et al. confirmed this association in a multicenter cohort of 803 patients with non-small cell lung cancer, with a median overall survival of 23.7 months (95% CI, 19.3-29.1) in patients with immune-related adverse events vs. 9.8 months (95% CI, 8.7-11.4) in those without (p < 0.001) [12]. In this patient, bullous pemphigoid preceded and accompanied the CR and may represent a marker of effective immune activation.

HPV-positive tumors exhibit a distinct immunological tumor microenvironment characterized by greater CD8+ T-cell infiltration, elevated presence of tertiary lymphoid structures, and expression of viral neoantigens, features collectively associated with enhanced immunogenicity and improved responsiveness to immune checkpoint inhibitors [5,6]. However, the tumor microenvironment of HNSCC is highly complex: multiple immunosuppressive cell populations, including tumor-associated macrophages, myeloid-derived suppressor cells, and regulatory T cells (Tregs), interact through cytokine networks that can limit effective antitumor immunity, even in HPV-positive disease [13]. The balance between these immunostimulatory and immunosuppressive forces is dynamic and may vary considerably between individual patients, potentially contributing to the wide variability in treatment response observed across the HPV-positive subgroup. In the present case, the sustained CR achieved despite low PD-L1 expression and extensive prior treatment suggests that additional immunological mechanisms beyond PD-L1-mediated signaling, possibly including CD8+ T-cell reactivation facilitated by the viral antigen burden and a favorable effector-to-suppressor cell ratio, may have contributed to durable tumor control.

It is important to acknowledge the inherent limitations of a case report, namely the inability to establish causal relationships and to generalize findings. The relative contribution of palliative radiotherapy and nivolumab to the CR cannot be determined with certainty. Furthermore, the absence of more detailed molecular data, such as tumor mutational burden or immune microenvironment analysis, limits interpretation of the underlying mechanisms. Finally, quality of life was assessed qualitatively during routine follow-up; validated patient-reported outcome instruments were not applied, which limits objective characterization of the patient's functional and symptomatic status.

This case reinforces the need to identify predictive biomarkers that may enable the selection of patients who could benefit from shorter durations of immunotherapy while maintaining prolonged disease control and contributes to the discussion regarding the possibility of treatment discontinuation in selected patients [4,7].

## Conclusions

We report a case of durable CR in a heavily pretreated HPV-positive oropharyngeal carcinoma, maintained for more than four years after nivolumab discontinuation. This case underscores the potential for long-term remission with immunotherapy, even in unfavorable clinical settings, and raises pertinent questions regarding optimal treatment duration, patient selection, and the role of immune-related adverse events as a potential favorable prognostic marker.
